# A Rare Case of Systemic AL Amyloidosis with Muscle Involvement: A Misleading Diagnosis

**DOI:** 10.1155/2018/9840405

**Published:** 2018-01-31

**Authors:** Fabrizio Accardi, Valentina Papa, Anna Rita Capozzi, Gian Luca Capello, Laura Verga, Cristina Mancini, Eugenia Martella, Roberta Costa, Laura Notarfranchi, Benedetta Dalla Palma, Franco Aversa, Vladimiro Pietrini, Giovanna Cenacchi, Nicola Giuliani

**Affiliations:** ^1^UO di Ematologia e CTMO, Azienda Ospedaliero-Universitaria di Parma and Department of Medicine and Surgery, University of Parma, Parma, Italy; ^2^Department of Biomedical and Neuromotor Sciences, Alma Mater University of Bologna, Bologna, Italy; ^3^Muscle and Nerve Histopathology Laboratory, Neurology Unit, Azienda Ospedaliero-Universitaria di Parma, University of Parma, Parma, Italy; ^4^Department of Diagnostic Medicine, Electron Microscopy Laboratory, IRCCS Fondazione Policlinico San Matteo and University of Pavia, Pavia, Italy; ^5^Pathology Unit, Azienda Ospedaliero-Universitaria di Parma, Parma, Italy

## Abstract

Muscle involvement in AL amyloidosis is a rare condition, and the diagnosis of amyloid myopathy is often delayed and underdiagnosed. Amyloid myopathy may be the initial manifestation and may precede the diagnosis of systemic AL amyloidosis. Here, we report the case of a 73-year-old man who was referred to our center for a monoclonal gammopathy of undetermined significance (MGUS) diagnosed since 1999. He reported a progressive weakness of proximal muscles of the legs with onset six months previously. Muscle biopsy showed mild histopathology featuring alterations of nonspecific type with a mixed myopathic and neurogenic involvement, and the diagnostic turning point was the demonstration of characteristic green birefringence under cross-polarized light following Congo red staining of perimysial vessels. Transmission electron microscopy (TEM) confirmed amyloid fibrils around perimysial vessels associated with collagen fibrils. A stepwise approach to diagnosis and staging of this disorder is critical and involves confirmation of amyloid deposition, identification of the fibril type, assessment of underlying amyloidogenic disorder, and evaluation of the extent and severity of amyloidotic organ involvement.

## 1. Introduction

Immunoglobulin light chain (AL) amyloidosis is the more frequent type of acquired amyloidosis.

The disease originates from a monoclonal misfolded light chain, produced by a plasma cell or B-cell clone, with a tendency to aggregation and tissue deposition leading to organ dysfunction. Mostly, it is a systemic disease, with a potentially generalized organ injury, but localized deposits are described [1]. Muscle involvement in AL amyloidosis is a rare condition, and the diagnosis of amyloid myopathy is often delayed and underdiagnosed. Amyloid myopathy may be the initial manifestation and may precede the diagnosis of systemic AL amyloidosis.

## 2. Case Presentation

Here, we report the case of a 73-year-old man who was referred to our center in November 2014 for a monoclonal gammopathy of undetermined significance (MGUS) diagnosed since 1999. He reported a progressive weakness of proximal muscles of the legs with onset six months previously. He did not present bone or muscle pain nor experienced limitations in instrumental activities of daily living. Laboratory data showed that blood count, electrolytes, including calcium, and renal and liver function were within normal range. A monoclonal component on *γ*-region on serum protein electrophoresis was described, equal to 2 gr/dl. Serum immunofixation was positive for IgG-*κ*, Bence Jones proteinuria was 244 mg in 24 hours, *κ* free light chains were 338 mg/L, and *λ* free light chains were 10.8 mg/L with an abnormal ratio equal to 31.3. A complete bone marrow examination and several imaging studies were performed. A bone marrow aspirate showed 30% of plasma cells restricted to *κ* chain by immunohistochemistry. Fluorescence in situ hybridization (FISH) analysis was negative for the presence of del(13q), del(17p), and chromosome 14 rearrangements. Conventional skeletal radiography excluded lytic lesions.

^18^FDG-PET did not display areas of increased uptake. A spine gadolinium-enhanced MRI detected normal bone marrow signals and two herniated discs at the lumbar and sacral levels (L4-L5 and L5-S1). The bone densitometry study revealed osteoporosis, and the patient was treated with vitamin D supplementation and bisphosphonates. Considering the absence of an event defining the disease as active, the plasma cell dyscrasia was classified as smoldering multiple myeloma (SMM).

During the follow-up period, the patient reported the appearance of pain and stiffness at the shoulders and hips and jaw claudication. Antinuclear antibodies (ANA) were negative, TSH was in normal range, creatinine phosphokinase (CPK) was 116 UI/L (normal value: 5–174), and B12 level was 162 pg/ml (192–1037). The mild B12 deficiency was corrected. Suspecting polymyalgia oral predinisone was started but without any clinical benefit therapy was interrupted. An electromyography revealed normal motor unit potentials.

**Figure 1:**
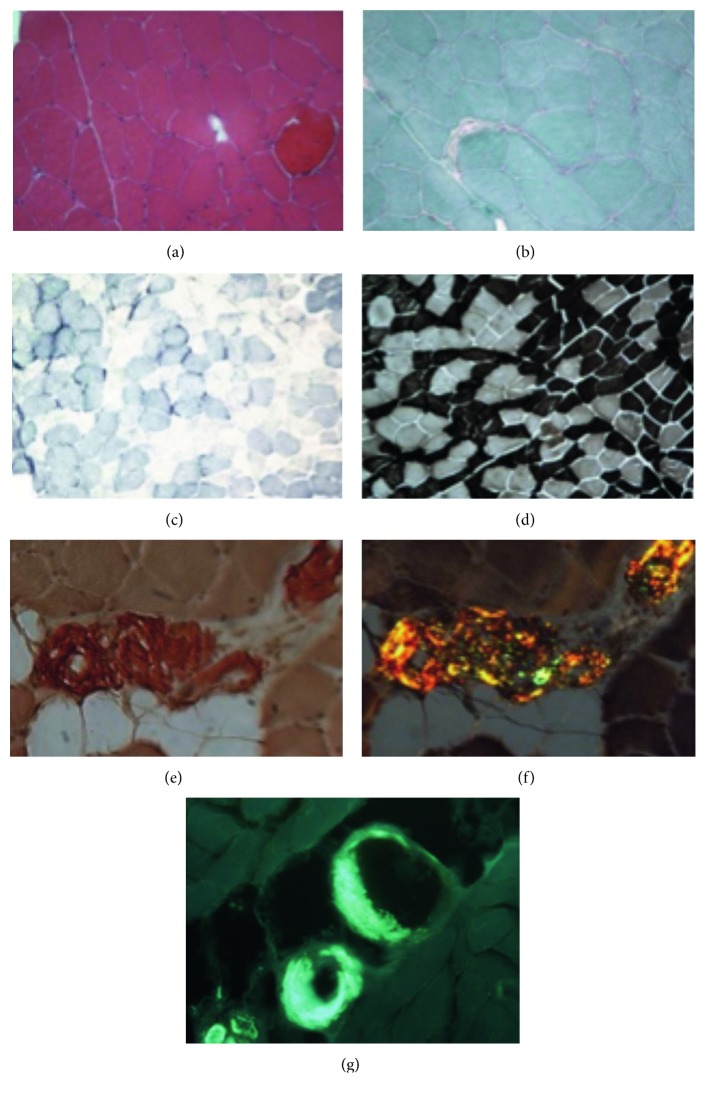
Muscle biopsy general features and the presence of amyloid substance. (a) Hematoxylin and eosin staining: the muscle cells show regular morphological characteristics with the exception of one hypercontracted cell; (b) Gomori staining: normal histological picture; (c) succinate dehydrogenase staining (SDH): the staining shows mild changes in myofibrillar texture; (d) ATPase pH 9.4: normal muscle fiber typing and distribution. Congo red staining without (e) or with polarized light (f): presence of amyloid around/inside the wall of perimysial vessels (×20); (g) Thioflavin S staining with fluorescence microscope: amyloid substance appears bright green in dark field. It completely includes the vessel wall of some perimysial arterioles (×20).

Because of the persistent symptoms, a clarifying left quadriceps muscle biopsy was taken on June 2016. It showed mild histopathology featuring alterations of nonspecific type with a mixed myopathic and neurogenic involvement (Figures 1(a)–1(d)). Considering the diagnosis of SMM, a light chain deposition could be suspected; the diagnostic turning point was the demonstration of characteristic green birefringence under cross-polarized light following Congo red staining of perimysial vessels (Figures 1(e) and 1(f)). In addition to the standard stainings, a differential diagnosis with other myopathies was performed by including histoenzymatic reactions. Moreover, the metabolic component was analyzed with the appropriate reactions, and any inflammatory aspects were explained by immunohistochemistry (data not shown). For further confirmation of the presence of amyloid in the wall of some muscle vessels, we also stained with Thioflavin S which showed the localization of amyloid in the same zones dyed with Congo red but in greater quantity (Figure 1(g)).

**Figure 2:**
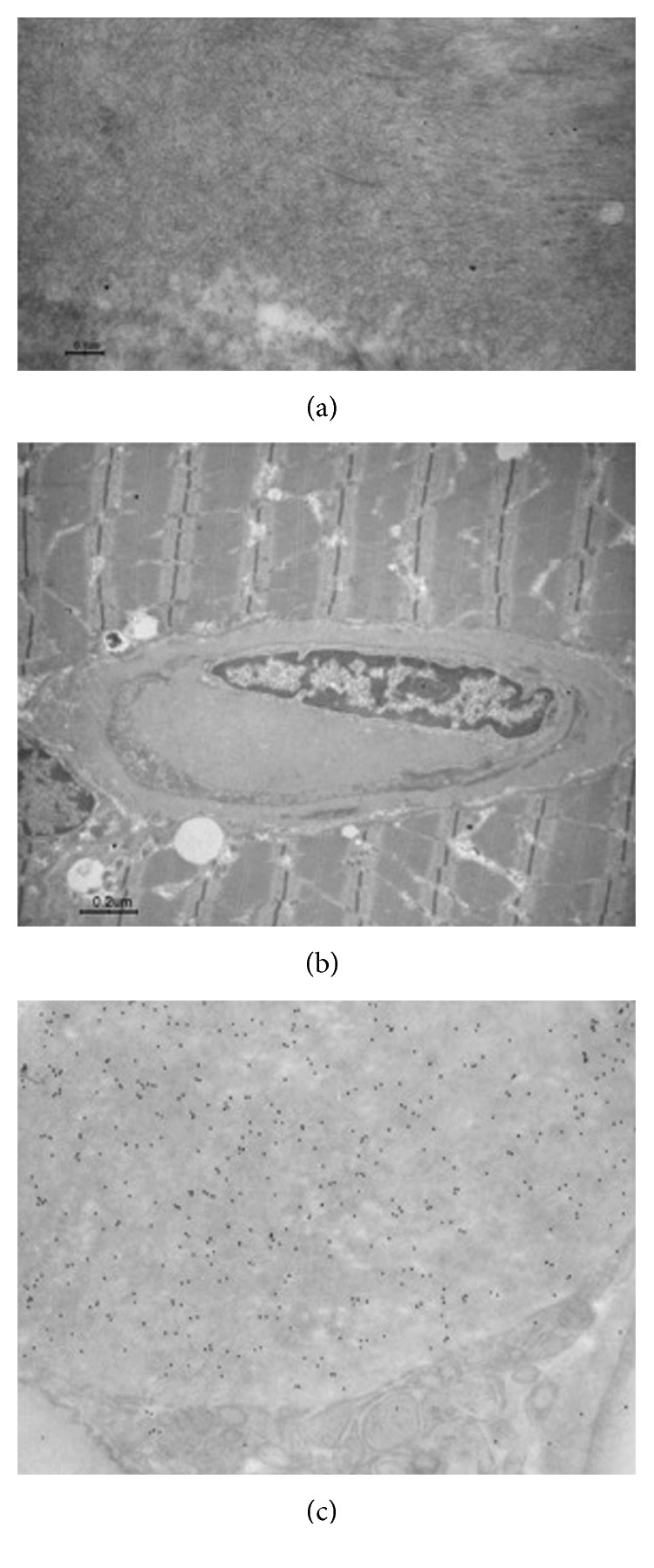
Presence of amyloid fibrils confirmed by electron microscopy. Muscle biopsy was routinely fixed in 2.5% glutaraldehyde in cacodylate buffer, postfixed in osmium tetroxide, and dehydrated and embedded in Araldite; thin sections were studied under Philips CM100 TEM fibrillary structures likely resembling amyloid fibrils in the perimysial pericapillary area (a) (bar = 0.1 *µ*m); an endomysial capillary lumen with a lightly thicker wall without amyloid fibrils (b) (bar = 0.2 *µ*m). Postembedding immunostaining with a polyclonal anti-kappa light chain antibody (Dako, 1 : 100) thin section was studied under a Jeol JEM-1400 Plus electron microscope (c).

**Figure 3:**
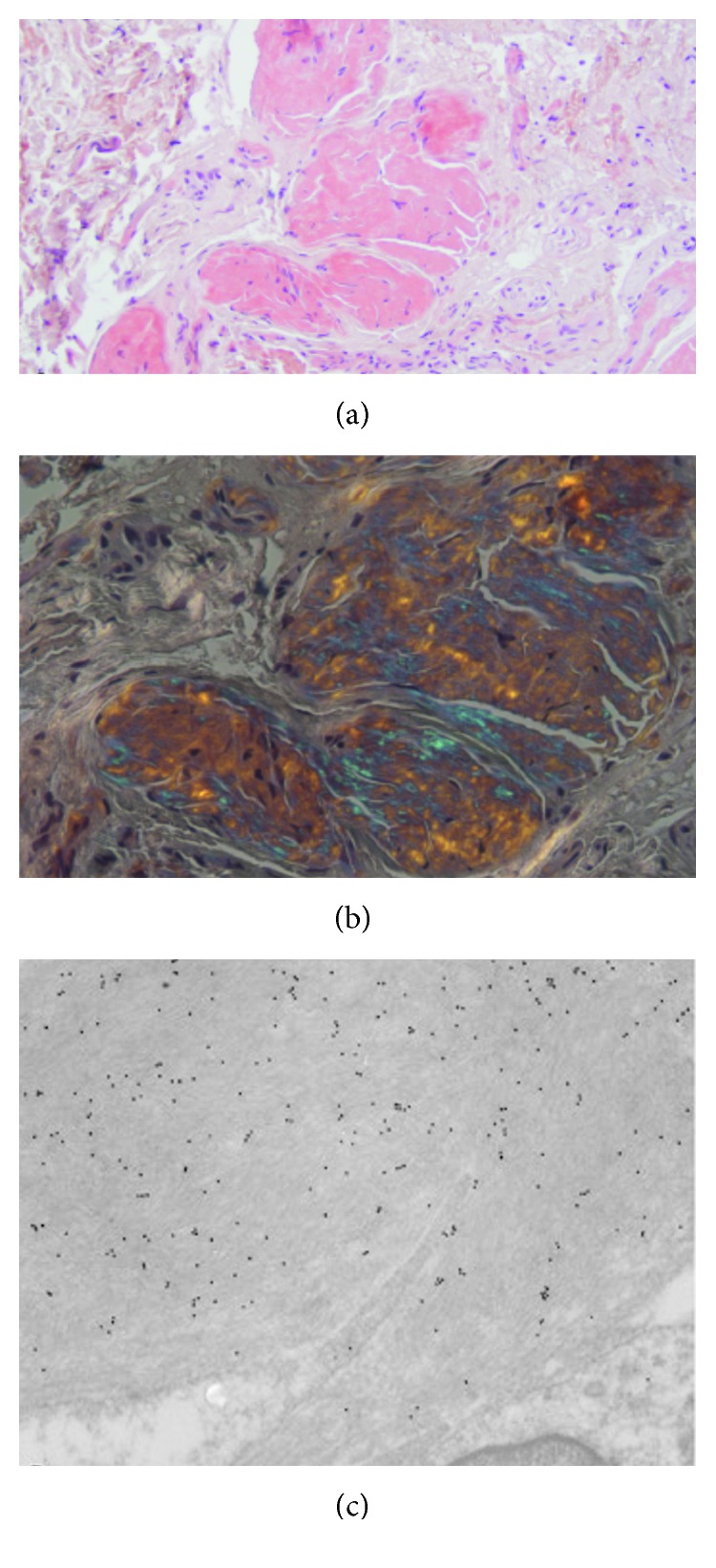
Presence of amyloid in the salivary gland. (a) Hematoxylin and eosin staining: some more dark areas between the muscle fibers are observed (×10). Olympus Bx51 optical microscope. (b) Congo red staining with polarized light: apple green birefringence. (c) Postembedding immunostaining: amyloid fibrils are intensely and specifically immunostained with anti-kappa light chain antibody (Dako, 1 : 100). Jeol JEM-1400 Plus electron microscope.

Transmission electron microscopy (TEM) confirmed amyloid fibrils around perimysial vessels associated with collagen fibrils (Figure 2(a)); endomysial capillaries only showed a thickened wall (Figure 2(b)). On muscle biopsy, immunoelectron microscopy showed a strong staining by polyclonal anti-kappa light chain antibody, thus identifying the fibril type (Figure 2(c)). The abdominal fat pad was negative for the presence of amyloid deposition; on the contrary, the obtained salivary gland biopsies were positive for Congo red staining (Figures 3(a) and 3(b)) as confirmed by immunoelectron microscopy (Figure 3(c)).

A biochemical reevaluation of the patient showed the lack of increase of cardiac serum biomarkers (BNP: 55 pg/ml, normal value: 0–100, and troponin I: <0.01 ng/ml, normal value: 0.01–0.06). Urinary albumin was negative, and a mild CPK increase was noticed. Echocardiography revealed normal left ventricular volume and thickness and a sigmoid-shaped ventricular septum. The systolic function was preserved. A diagnosis of AL amyloidosis with muscle involvement was made, and a treatment with cyclic oral melphalan and dexamethasone was started. The patient is currently under treatment.

## 3. Discussion

The identification of the muscle as the unique site of amyloid accumulation is a very rare event. In a recent report on 3434 patients diagnosed with AL amyloidosis, 51 (1.5%) had a muscle biopsy positive for amyloid deposition. Among these, amyloid muscle involvement was isolated in 11 patients (22%). Common presenting symptoms of patients with amyloid myopathy are muscle weakness, myalgia, skeletal pseudohypertrophy, dysphagia, macroglossia, jaw claudication, and hoarseness [2]. Our patient showed progressive muscle weakness of lower limbs with subsequent appearance of pain and stiffness at the shoulders and hips and jaw claudication without marked biochemical signs of muscle involvement.

The conclusive diagnosis was systemic AL amyloidosis with myopathic involvement as amyloid deposits were also detected in the salivary gland. However, the myopathy was the only clinical manifestation of the disease because any other symptoms and signs of systemic AL amyloidosis involvement, like cardiac dysfunction or renal proteinuria, were not present. In a recent study, Liewluck and Milone reported that, in nearly 70% of patients with AL amyloidosis muscle involvement, myopathy was the only sign at the clinical presentation [3]. AL systemic amyloidosis-associated myopathy differs from pure isolated amyloid myopathy caused by other amyloidogenic protein subtypes for some clinical characteristics such as older age at onset and less frequent CPK elevation [3].

In this case, the time from the first muscular symptom onset and amyloidosis diagnosis was 25 months. Diagnostic delay of amyloidosis is often reported, as recently described in a patient experience survey where 37.1% of interviewed patients received the correct diagnostic formulation after more than one year from the initial symptoms [4].

In Mayo Clinic case series of biopsy-confirmed muscle AL amyloidosis, the median time from the first disease manifestations and diagnosis was 23 months [2]. Unlike other amyloid proteins, in AL amyloidosis, any organ can be affected by amyloid deposition, and well-defined criteria of organ involvement were described [5, 6]. The diagnostic pitfall may be of particular relevance when the amyloid deposition and the resulting organ damage involve, solely, a less common site such as the striated muscle.

In 19 of 79 cases reviewed by Chapin et al., the initial muscle biopsy was unable to identify the presence of amyloid deposition [7]. This was due to different reasons such as the lack of involvement of that specific muscle district by a “random” biopsy or the absence of Congo staining [8]. In agreement with this observation, Muchtar et al. reported that a different muscle disease was diagnosed in about 40% of the patients before the histological revision [2]. The delay in diagnosis is an important issue and increases the risk of disease aggravation, particularly with cardiac involvement, that determines the disease stage and prognosis [9, 10].

Interestingly, three main clinical presentation patterns of amyloid myopathy are described in literature case series and case reports [2, 7, 11]. The first group reveals the distinctive feature of skeletal pseudohypertrophy with palpable nodules in muscles and “wood-like” atypical consistency often associated with macroglossia. The second group includes patients affected by muscle weakness, predominantly proximal, possibly accompanied by atrophy without other signs of amyloid tissue deposition. The “atrophic form” represents a clinical challenge because the diagnosis is difficult and should take into account all possible differential diagnoses of myopathy [12]. The third group is a mixed clinical phenotype.

The pathophysiological mechanism of muscle fiber injury is poorly clarified. In muscle amyloidosis, the fibril deposition is universally observed on endomysial and perimysial vessels, as in our case, suggesting as a possible mechanism the chronic ischemia derived from endothelial damage [13]. Less frequently described are denervation atrophy and necrotic fibers with regeneration signs [2, 7]. An increase in protein synthesis and cell fusion of human myoblasts in culture was reported by Delaporte et al. after the exposure to a purified kappa chain serum of a patient affected by muscle pseudohypertrophy [14]. A striking characteristic of AL amyloidosis is the enormous interpatient variability in the protein structure and tissue tropism that was also observed in amyloid proteins derived from the same variable light chain gene. A little change in the amino acid sequence acquired during hypersomatic mutation of immunoglobulin genes may greatly influence the specificity and type of organ damage [15].

In the present case, chemotherapy was promptly started after diagnosis as the therapeutic goals in AL systemic amyloidosis should be the eradication of the underlining clone, the suppression of the production of the dangerous light chain, and the prevention of further organ damage [1].

The accurate characterization of muscle biopsy was essential for the correct diagnosis.

The adoption of Congo red staining, in histopathological examination of an undetermined myopathy, represents an important teaching point in order to avoid a failure in diagnosis. An unexplained muscle disorder in patients with monoclonal gammopathies, by including MGUS, should evoke, among other possible diagnoses, the suspicion of amyloid deposition, and the analysis of biomarkers of early amyloid organ involvement, like albuminuria and cardiac markers, together with abdominal fat or salivary gland biopsies should be performed.

This report suggests that a multidisciplinary approach is the cornerstone of the diagnostic work-up to recognize the rare amyloid myopathy. A stepwise approach to diagnosis and staging of this disorder is critical and involves muscle biopsy, confirmation of amyloid deposition, identification of the fibril type, assessment of underlying amyloidogenic disorder, and evaluation of the extent and severity of amyloidotic organ involvement.
